# Nano-nutraceuticals from spinach-iron oxide as a green remedy for iron deficiency

**DOI:** 10.1016/j.crfs.2025.101243

**Published:** 2025-11-14

**Authors:** Aisha Tariq, Huma Umbreen, Shafaqat Ali, Razia Noreen, Zanab Qasim, Santiago P. Aubourg, Meshari A. Alsuwat, Muhammad Abdul Rahim, Eliasse Zongo, Mohamed Fawzy Ramadan

**Affiliations:** a Department of Nutritional Sciences, Government College University Faisalabad, Faisalabad, Punjab, Pakistan; b Department of Environmental Sciences, Government College University Faisalabad, Faisalabad, Punjab, Pakistan; c Department of Biochemistry, Government College University Faisalabad, Faisalabad, Punjab, Pakistan; d Marine Research Institute (CSIC), Street: E. Cabello, 6, 36208-Vigo, Spain; e Department of Basic Sciences, College of Nursing, Taif University, P.O. Box 11099, Taif, 21944, Saudi Arabia; f Department of Food Science & Nutrition, Faculty of Medicine and Allied Health Sciences, Times University, Multan, Punjab, 60700, Pakistan; g Laboratory of Research and Teaching in Animal Health and Biotechnology, Nazi Boni University, 01 BP 1091, Bobo-Dioulasso, Burkina Faso; h Department of Clinical Nutrition, Faculty of Applied Medical Sciences, Umm Al-Qura University, Makkah, 24231, Saudi Arabia

**Keywords:** Spinach, Nanotechnology, Iron deficiency anemia, FTIR, XRD, Diet study

## Abstract

Iron deficiency anemia (IDA) is a nutritional metabolic condition that effects infants, children, even adults, both men and women. Individuals with IDA have inadequate iron intake or impaired absorption of iron. The main cause of IDA is the low bioavailability of iron from conventional means, diet and supplementation, even if present in an ambient amount. The major objective of this study was to increase the bioavailability of iron using green technology. For this purpose, iron oxide nanoparticles were fabricated using iron-rich spinach leaves extract. The characterization of the particles was done by Fourier Transform Infrared Spectroscopy (FTIR), X-ray Diffraction (XRD), Ultraviolet–Visible spectroscopy (UV–Vis) and Scanning Electron Microscope (SEM). For bio-evaluation of these green synthesized nanoparticles, male albino rats (n = 45) weighing 120–150 g were used in the study. Iron deficiency anemia was induced by using phenyl-hydrazine injection (40 mg/kg) in all rats except for the negative control group. Anemic rats were subdivided into four groups: one positive control and three treatment groups (spinach powder (7 % of feed), iron oxide nanoparticles (4 mg/kg BW/day), ferrous sulfate (4 mg/kg BW/day). The duration of the intervention period was 21 days. Hematological measures were taken to check the treatment effects of iron levels. The findings of the study demonstrated that hemoglobin, serum iron and serum ferritin levels in intervention groups were found to be significantly improved with p = 0.001. The present study concluded that spinach-iron oxide nanoparticles could be an effective approach in treating iron deficiency anemia and may be used for novel functional foods and in the medicinal field.

## Highlights

• Spinach-based iron oxide nanoparticles were synthesized and characterized.
• For bio-evaluation study in rats, IDA was induced using phenyl-hydrazine.
• Iron nano-nutraceutical treatment showed better hematological levels.
• Nano-nutraceutical treatment outperformed commercial iron supplements.
• Liver and renal function tests showed no adverse effects in rats.

## Graphical abstract

**Figure:**
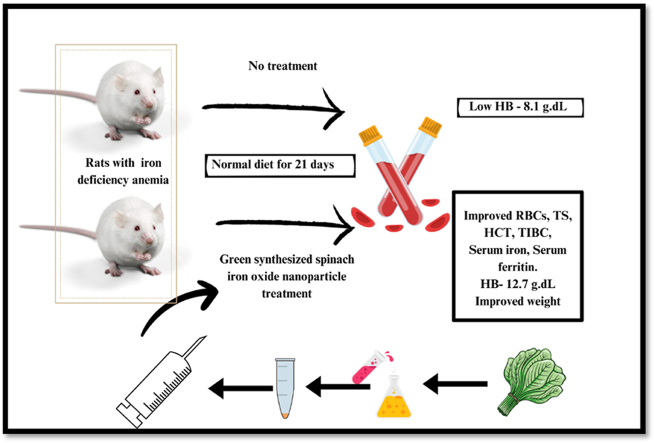

## 1 Introduction

Anemia is red blood cell (RBC) or hemoglobin (Hb) deficiency and has been identified as a major health concern by World Health Organization (WHO) particularly in underdeveloped nations. Anemia is a symptom of an underlying issue rather than a disease. The most prevalent type of anemia is iron deficiency anemia (IDA), which may be caused by low intake of iron, low absorption, excessive bleeding, parasitic infections, gastric and duodenal ulcers/cancers and can affect newborns, children and adults of both genders (Arora and Kaur, 2020; Bathla and Arora, 2021; Kumar et al., 2022; Shubham et al., 2020). The conventional methods of treating nutritional iron deficiency in populations are diet modification and food fortification with ferrous and ferric iron salts (ferrous sulfate, ferrous gluconate, ferric citrate and ferric sulfate) (Salaheldin and Regheb, 2016). Despite being extensively prescribed, conventional iron has not only undesired effects on digestion and taste but has also poor gastrointestinal absorption and lower bioavailability. Thus, it is much needed to develop cost effective, ecofriendly yet innovative iron sources with higher bioavailability (Breymann and Auerbach, 2017; Kumari and Chauhan, 2022).

Spinach (*Spinacia oleracea*), a member of the Amaranthaceae family is consumed all over the world and is rich in polyphenols and flavonoids that have shown anti-inflammatory, anti-mutagenic and anti-cancer effects in living bodies (Fayyaz et al., 2022). It is also considered as a good source of iron, riboflavin, niacin and folic acid along with vitamins, minerals and dietary fibers; however, it presents a low availability in human body (Patricia et al., 2014). The advancement of nanotechnology resolves this issue as nanocomposites can address the issue of low availability and absorption of biologically important materials and are being employed as adjunctive therapies for a growing range of disorders in the medical sector (Magne et al., 2023). In the last decade, biosynthesis of metal oxide nanoparticles has received increasing attention due to the potential properties including optical, electronic, mechanical, magnetic, chemical and biomedical (Kulkarni and Thakur, 2018). Notably, nanoparticles are more efficient as they provide targeted drug delivery, improve bioavailability and enhance drug stability (Zahin et al., 2020). It is found as a convenient method in the health sector because of the unique characteristics of nanoparticles (i.e., tiny size and high surface-to-volume ratio), however their chemical synthesis is major hindering factor (Manocha et al., 2022). Since spinach is a rich iron source, it is anticipated that utilizing only a low dosage of iron oxide nanoparticle green synthesized with spinach extract would not only boost the bioavailability of iron but would also address the adverse effects of its supplements on gastrointestinal tract along with compensating toxic nature of chemically synthesized nanoparticles (Elshemy, 2018).

The use of spinach leaf extract for the one-step green synthesis of iron oxide nanoparticles eliminates the need for high pressure or temperature and is therefore both environmentally benign and energy-efficient. Consequently, this study presents innovative and workable solution to the gap in the need for techniques in iron-based nutraceuticals, which are not only efficient as compared to usually used therapy but are also environment friendly and sustainable. Therefore, the present study aimed to synthesize iron oxide nanoparticles through green method using spinach leaves extract and to assess and compare the impact of spinach-based iron oxide nanoparticles with spinach powder and a commercial iron supplement on blood markers of iron deficient rats. Furthermore, dose used for bio-evaluation of these spinach-based green synthesized iron oxide nanoparticles to mitigate iron deficiency anemia (IDA) offers a viable and environmentally acceptable substitute for traditional iron supplements (with low efficiency and multiple side effects), thus presenting a novel application in biomedical research about the most prevalent deficiency disorder of the vulnerable population.

## 2 Materials and methods

The present study was conducted in Government College University Faisalabad, Pakistan. The study was comprised of two phases: the synthesis and characterization of spinach-iron oxide nanoparticles, and the bio-evaluation of those particles on iron-deficient rats.

### 2.1 Synthesis of iron oxide nanoparticles

**Fig. 1:**
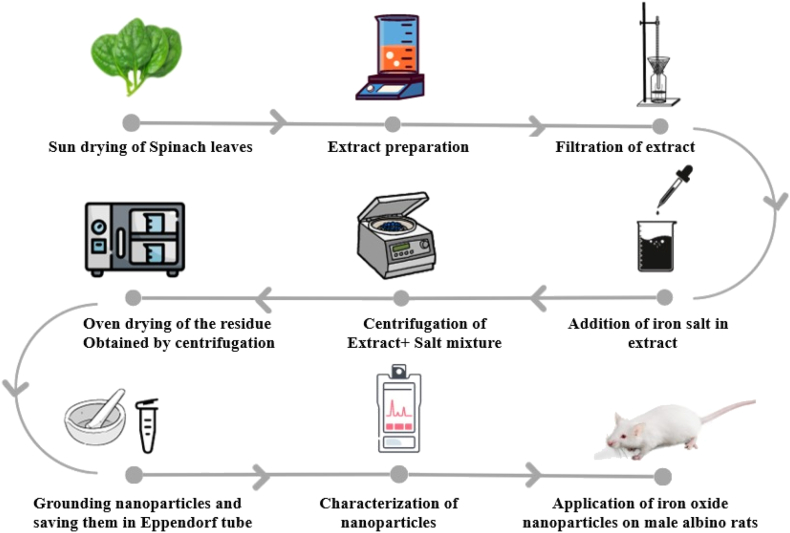
The mechanism for synthesis of nanoparticles utilizing plant extract.

Spinach (*Spinacia oleracea*) leaves were purchased from a local market. Ferrous-sulfate supplement was purchased from the medical store. The research facilities and equipment from the Department of Nutritional Sciences and Department of Environmental Sciences, GCUF, were used. All chemical reagents including Iron (III) nitrate 9-hydrate, methanol, ethanol and blood assay kits were purchased from Sigma Aldrich and Randox Laboratories (Antrium, UK). For extract preparation, spinach leaves were shade dried, weighted 50 g of dried leaves and boiled in a beaker on a hot plate for about 90–100 min at 90 °C in 500 mL distilled water. The obtained extract was first strained and then filtered using Whatman filter-paper (Obidah Abert et al., 2022). Iron oxide nanoparticles (IONPs) were prepared by using 0.1 M solution of iron (III) nitrate 9-hydrate (Fe(NO_3_).9H_2_O) salt (Di Iorio et al., 2019). The extract-salt solution was then left for about 1 h and then was centrifuged for 10 min at 5000 rpm with a fixed angel rotor (Megafuge 16 Small Benchtop Centrifuge, Thermo Fisher Scientific, Dreieich, Germany) at 6755×*g*. The supernatant was discarded and the residue pellet obtained after centrifugation was dried in hot oven at about 80 °C for 2–3 days. When dried properly, the pellet was ground into fine powder with the help of motor-pestle and saved in the eppendorf tubes until further use. Fig. 1 explains the mechanism of synthesis of nanoparticles using spinach leaves extract and its further utilization.

### 2.2 Characterization of nanoparticles

The spinach-iron oxide NPs were characterized by evaluating their shape, size, morphology and surface area, using various characterization techniques.

The type of associated functional groups of plant extract with nanoparticles was determined by Fourier Transform-Infrared spectroscopy (FTIR), carried out on dried sample, which were ground into fine powder using pestle and mortar to ensure homogeneity. To get the spectra FTIR spectrometer (Catrry-630, Santa Clara, California, USA) was used and range was set to 640–4000 cm^−1^. The samples were placed onto a flat glass plate at room temperature. Another plate was placed on top and a quarter turn made to get a good even film. The plate was placed in the sample holder and the spectrum was run at a resolution of 4 cm^−1.^ The absorption peaks were obtained according to vibrational frequencies of functional groups.

Similarly, the crystalline structure of the samples was analyzed using an X-ray Diffraction (XRD). For the purpose the dried and ground sample as in FTIR was used. An X-ray Diffractometer (Bruker-D8. Advance X-ray Diffractometer, Karlsruhe, Germany) with Cu Kα radiation source was set at angle of 2θ in the range of 5°–70° with wavelength of 0.154 nm and scan rate was adjusted to 0.02° min^−1^. For optimal texturing, each sample was placed in a slot, pressed with frosted glass and then XRD pattern was observed.

Furthermore, the structures, size distribution and the surface morphology of green synthesized particles were depicted using a Scanning Electron Microscope (SEM) (Amray-3300, FE-SEM, Bedford, MA, USA). For the purpose, the dried sample was securely fastened on the sample holder and was inserted into sample chamber. The accelerating voltage was set to low (5 kV) to avoid any potential damage to green components of nanoparticles. A secondary electron detector was used to check surface morphology, while working distance was optimized for better focus. Starting from lower magnification, the suitable area with even distribution of nanoparticles was sought and magnification was increased to capture image at 1, 3 and 5 μm resolution. The captured images were used to determine morphology and size distribution of the nanoparticles.

While, to confirm the formation and optical properties of the nanoparticles, the UV–visible spectra were recorded using UV–visible spectrophotometer (T-80 UV–Visible Spectrometer, Leicestershire, UK). The iron oxide nanoparticles were suspended in distilled water to make a colloidal solution, while the distilled water was used as blank. The wavelength of the spectrophotometer was set in a broader range of 200–800 nm and the mode was set to absorbance. First the absorbance was checked for blank then the sample was added to the cuvette and broad range of absorbance spectrum was obtained. The peak wavelength was identified which presents maximum absorption (*λ*_max_). The optical band gap (Eg) was determined by extrapolating on X-axis of a Tauc plot (Smital et al., 2020).

### 2.3 Bio-evaluation study

#### 2.3.1 Study design

At the beginning of the trail a total of 45 male albino rats were used in this research study. The weight of all the rats was within the range of 120–130 g. All rats were divided into 5 groups at random and named G_1_, G_2_, G_3_, G_4_ and G_5,_ respectively (Table 1). Each group contained 3 replicates and each replicate contained 3 rats which were kept in wire cages. Rats were acclimated for five days prior to the experiment, and given unlimited access to food and water. The housing was run on 12-h cycles of light and darkness. An iso-caloric and iso-nitrogenous AIN-93-G based diet was given to all the groups. Anemia was introduced in rats (except for the negative control group) by 40 mg/kg of phenyl-hydrazine injected intraperitoneal for two consecutive days. To confirm anemia, red blood cells and hemoglobin tests were done (Abdo et al., 2021). The study's approval was obtained from the Ethical Review Committee of the University (Ref No. ERC/211). The experimental details and animal care was adopted in line with ARRIVE guidelines (Percie du Sert et al., 2020). The doses (Table 1) for the treatment groups were administered orally by gastric gavage.

**Table 1:** Treatment plan for all groups.

Group	Condition	Treatment
**G_1_**	Normal (negative control)	No treatment
**G_2_**	Anemic (positive control)	No treatment
**G_3_**	Anemic	Spinach powder (7 % of the feed)
**G_4_**	Anemic	Spinach-Iron oxide nanoparticles (4 mg/kg B.W./day)
**G_5_**	Anemic	Iron supplementation (4 mg/kg B.W./day)

#### 2.3.2 Sampling

For the collection of blood samples at the end of research, dissection of rats was done after euthanizing them. The blood was collected in the vials, centrifuged and serum was preserved at −20 °C. The procedure was done according to the guidelines for euthanizing animals in the research setting to ensure that it is done as painlessly and humanely as possible.

#### 2.3.3 Biochemical analysis

Complete blood count (CBC) was conducted by using Automatic Hematology Analyzer counter (Mythic-3CRP, Germany Diadnostics). To have further insight into the blood parameter related to iron deficiency anemia, different determinations were carried out such as serum iron concentration, serum ferritin concentration, serum transferrin concentration, total iron binding capacity (TIBC) and transferrin saturation (TS) by using the methods described by He et al. (2019) The safety analyses through liver and kidney functions tests were carried out to check any damage of the spinach-iron oxide nanoparticles and/or other treatments used in the exposed treatments (Smital et al., 2020). Liver function tests (LFT), such as Alanine amino transferase (ALT), Aspartate aminotransferase (AST), direct and indirect bilirubin, and Renal function tests (RFT), such as Creatinine and Blood Urea Nitrogen (BUN), were done to investigate the organs state after treatment (El-Bahr et al., 2021).

#### 2.3.4 Physical parameters

The physical parameters including food and water intake (daily basis) and body weight (weekly basis) of all the experimental groups were measured throughout the experiment time. By using these parameters, the feed efficacy ratio (FER) of every experimental group was calculated. FER is the simplest way to check the growth performance analysis of the animals such as rats (Rogaska et al., 2020). The feed efficiency ratio was obtained by dividing the average weight gain (g) of a group divided by the feed intake (g) of the group using equation (1) (Abdel Fattah et al., 2020);

(1) $$FER=\frac{Weight\;gain/day\;\left(g\right)}{Feed\;Intake/day\;\left(g\right)}$$

#### 2.3.5 Statistical analysis

The study was carried out in triplicate. Additionally, all the readings were also taken in triplicate. Minimum, maximum and quartiles for feed efficiency ratio were calculated to determine the interquartile range and MS excel was used to draw the Box plot. For other parameters data obtained were expressed as mean ± standard deviation (SD). The least significant difference (LSD) test was employed to compare between the various experimental groups after one-way ANOVA data analysis. The statistical software for the social sciences (SPSS) version 21 was used to statistically analyze the data. The values with level of significance at p ≤ 0.05 were considered statistically significant (Fathy et al., 2019).

## 3 Results

### 3.1 Functional groups detection

**Fig. 2:**
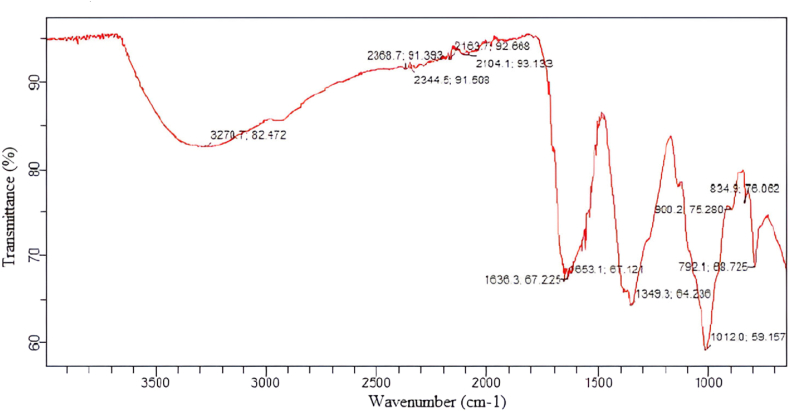
FTIR spectra of spinach-iron oxide nano-particles.

Fourier Transform-Infrared spectra (FTIR) of spinach-iron oxide nanoparticles synthesized by using spinach leaves extract in the 640- 4000 cm^−1^ wavenumber range is shown in Fig. 2. Among characteristic peaks, the hydroxyl and amid groups at ∼3270 cm^−1^ and 1636 cm^−1^, respectively, can be observed in the IR spectra of the synthesized spinach-iron oxide nanoparticle sample. From these IR spectra, it is apparent that the hydroxyl and amino groups found in proteins and carbohydrates exhibit the capability to serve dual roles in both reducing and stabilizing iron oxide nanoparticles. The peak situated at ∼1012 cm^−1^ is related to C = N groups, whereas the peak at 1349 cm^−1^ also showed C = N groups but with vibrations of the tetrapyrrole ring by iron oxide nano-particles. Vibration around 1653 cm^−1^ verified the C = C groups; meantime, C-H, O-H and C=O signals were observed at 1100-700 cm^−1^. The absence of any extra peaks in the FTIR spectrum indicated that the synthesized material is pure.

### 3.2 Structural examination

**Fig. 3:**
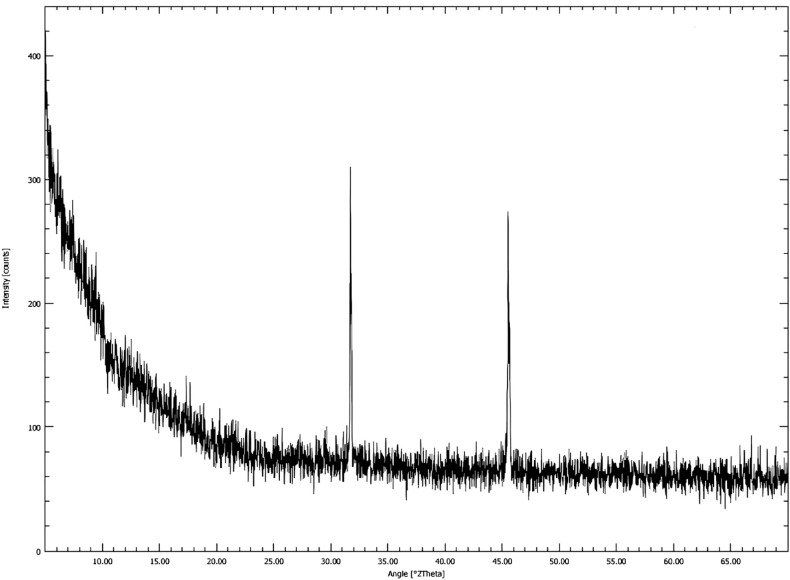
XRD pattern of spinach-iron oxide nano-particles.

The crystal structure of nanoparticle was analyzed by XRD within the range of 5°–70° angle. Thus, Fig. 3 shows the XRD pattern of the green synthesized spinach-iron oxide nanoparticle. The pattern was compared with reference to the standard XRD pattern. One of the most intense peaks is observed at 2θ ∼31.7° and the other most intense peak can be seen at 2θ 45.5°.

### 3.3 Morphological features

**Fig. 4:**
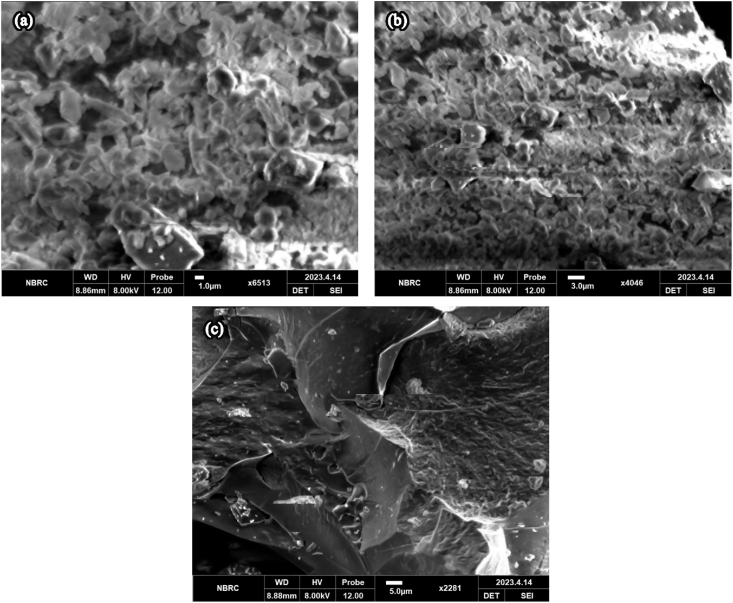
(a) SEM results of spinach-iron oxide nano particles at 1.0 μm resolution, (b) SEM results of spinach-iron oxide nano particles at 3.0 μm resolution, (c) SEM results of spinach-iron oxide nano particles at 5.0 μm resolution.

The morphology of the spinach-iron oxide nanoparticles, prepared by green synthesis using spinach leaves extract, were studied by SEM at various magnifications. The highly magnified images of the sample have been shown in Fig. 4. Fig. 4 (a) showed a blurred scanning electron microscope image of iron nano particles with the resolution of 1.0 μm, whereas Fig. 4b showed a somewhat clear image of the particles with the resolution of 3.0 μm. Similarly, image shown in Fig. 4c is clearer with the scale bar 5.0 μm. These micrographs of green synthesized iron nanoparticles exhibit clustered topography and variable surface morphology in terms of shape and size, which confirms its amorphous nature.

### 3.4 Optical analysis

**Fig. 5:**
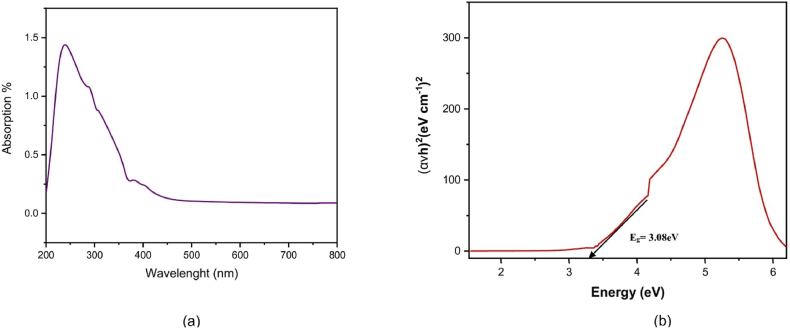
(a) Absorption characteristics in UV–Visible range for Spinach-iron oxide nano-particles (b) UV–Visible Transmission Spectrum of Spinach-iron oxide nano-particles.

The absorption spectrum of spinach-iron oxide nano particles is shown in Fig. 5 (a). The range of the examined spectrum is 200 nm–800 nm. From ∼225 to ∼275 nm, a broad absorption band was observed throughout the spectral range. The optical band gap of spinach-iron oxide nano particles was calculated by using the Tauc relation shown in Fig. 5 (b).

### 3.5 Feed efficiency ratio

**Fig. 6:**
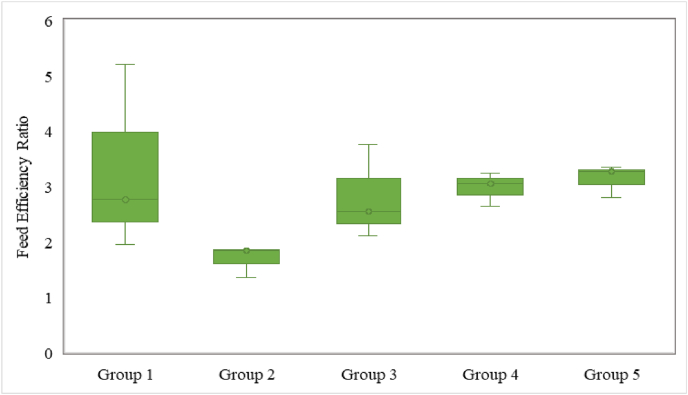
Feed efficiency ratio (FER) of different rat groups.

The physical parameters including feed intake, water intake and weight changes of all the rat's groups were measured regularly and their values were used to calculate feed efficiency ratio (FER). Fig. 6 showed the weekly feed efficiency ratio of all the groups, which represents significant difference (p ≤ 0.05) between the groups. It showed that the most increasing trend was observed in the negative control group. In contrast, the positive control group showed the lowest FER with no significant difference (p > 0.05) within the group during the treatment period.

### 3.6 Hematological parameters

The data shown in Table 2 indicate the effect of 21 days' treatment of spinach-based iron oxide nanoparticles. There was a significant reduction in hemoglobin concentration, hematocrits (HCT), red blood cells (RBCs), mean corpuscular volume (MCV) and mean corpuscular hemoglobin concentration (MCHC) in the positive control group (G_2_) as a result of induced iron deficiency anemia when compared to the normal rats group. Nano particles corresponding to the group G_4_ (namely, green synthesized Spinach-iron oxide) showed the most effective results on hemoglobin value in the treatment groups. The results showed that supplementation with spinach powder in G_3_ did not cause a significant (p > 0.05) change in hemoglobin concentration (9.35 ± 0.49 g/dL), hematocrits (HCT), RBCs (5.67 ± 0.39 cells/mcL), mean corpuscular volume (MCV) and mean corpuscular hemoglobin concentration (MCHC) as compared to the negative control group. Meanwhile, green synthesized Spinach-iron oxide nanoparticle's treatment group showed the most effective results on hemoglobin concentration (12.76 ± 0.54 g/dL) in the treatment groups. However, the treatment groups given iron supplement also showed significant effect on HB (11.03 ± 0.46 g/dL) when compared to the positive control (G_2_), but the results are less pronounced than Spinach-iron oxide nanoparticles. Similarly, it is also observed that results of HCT (44.37 ± 0.78 %), RBCs (7.45 ± 0.25 cells/mcL), MCV (84.9 ± 0.83 ft) and MCHV (33.08 ± 1.00 g/dL) in serum of Spinach-iron oxide nanoparticles group were found to be similar to those of the negative control group (G_1_). Furthermore, it was also observed that results of Spinach-iron oxide nanoparticles group were even better than those corresponding to the pharmaceutical supplement (G_5_) as well as spinach powder group (G_3_) in case for HCT, RBCs, MCV and MCHV (Table 2).

**Table 2:** Concentrations of biochemical parameters, liver function test and renal function test in blood (Mean ± SD) of different study groups.

	G_1_	G_2_	G_3_	G_4_	G_5_
**Hematological Parameters**					

**HB (g/dL)**	14.12 ± 0.92^a^	8.11 ± 0.37^e^	9.35 ± 0.49^d^	12.76 ± 0.54^b^	11.03 ± 0.46^c^
**HCT (%)**	48.11 ± 2.57^a^	27.66 ± 1.58^e^	37.18 ± 1.83^d^	44.37 ± 0.78^b^	42.32 ± 1.36^c^
**RBCs (cells/mcL)**	7.46 ± 0.36^a^	3.37 ± 0.19^d^	5.67 ± 0.39^c^	7.45 ± 0.25^a^	7.04 ± 0.29^b^
**MCV (fl)**	90.15 ± 1.52^a^	70.07 ± 4.43^d^	78.2 ± 2.26^c^	84.9 ± 0.83^b^	79.82 ± 1.52^c^
**MCHC (g/dL)**	34.18 ± 0.71^a^	22.46 ± 2.03^c^	28.12 ± 5.33^b^	33.08 ± 1.00^a^	32.82 ± 1.07^a^

**Liver Function Tests**					

**AST (U/L)**	26.44 ± 8.04^c^	45.22 ± 10.10^a^	35.44 ± 6.59^b^	29.88 ± 8.89^bc^	30.77 ± 9.44^bc^
**ALT (U/L)**	37.00 ± 11.82^c^	65.55 ± 8.81^a^	48.11 ± 5.73^b^	44.88 ± 6.99^bc^	47.77 ± 10.59^b^
**Total bilirubin (mg/dL)**	0.30 ± 0.07^b^	1.32 ± 0.14^a^	0.57 ± 0.40^b^	0.45 ± 0.31^b^	0.50 ± 0.38^b^
**Direct bilirubin (mg/dL)**	0.18 ± 0.10^b^	0.38 ± 0.16^a^	0.18 ± 0.12^b^	0.16 ± 0.10^b^	0.21 ± 0.09^b^
**Indirect bilirubin (mg/dL)**	0.50 ± 0.23^b^	0.83 ± 0.39^a^	0.53 ± 0.20^b^	0.52 ± 0.19^b^	0.56 ± 0.15^b^

**Renal Function Tests**					

**Creatinine (mg/dL)**	0.77 ± 0.13^b^	1.57 ± 0.31^a^	0.85 ± 0.22^b^	0.86 ± 0.20^b^	0.90 ± 0.22^b^
**BUN (mg/dL)**	13.33 ± 5.63^b^	21.33 ± 6.67^a^	16.22 ± 3.45^b^	14.33 ± 3.50^b^	16.44 ± 2.65^b^

^a-e^ Different letters in a row show significant difference at *p* ≤ 0.05, Hb = Hemoglobin; HCT= Hematocrit; RBCs= Red blood cells; MCV = mean corpuscular volume; MCHC = Mean corpuscular hemoglobin concentration; AST = Aspartate aminotransferase; ALT = Alanine aminotransferase; BUN= Blood urea nitrogen.

**Fig. 7:**
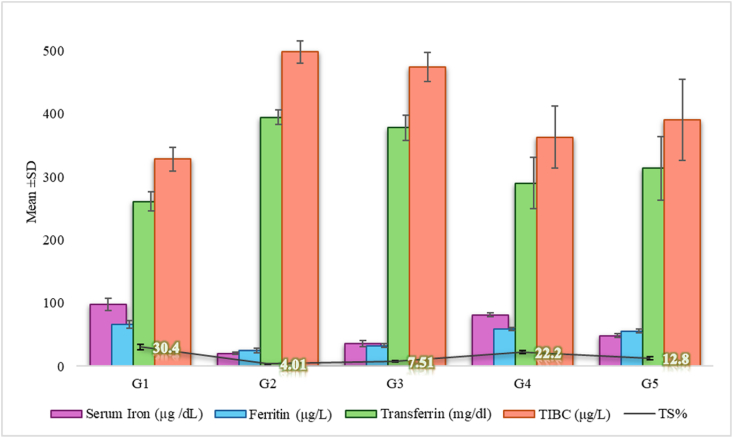
Comparative analysis of Serum parameters (Serum iron (μg/dL), Ferritin (μg/L), Transferrin (mg/dL), TIBC (μg/L)) with Transferrin Saturation % (TS%) overlay, error bar representing the Standard Deviation.

With respect to iron deficiency anemia, the present study focused on iron related parameters of the serum. Thus, Fig. 7 showed a significant reduction in serum iron (20.33 ± 2.17 μg/dL), serum ferritin (24.88 ± 3.40 μg/L) and a significant (p ≤ 0.05) increase in serum transferrin and total iron binding capacity TIBC (498.67 ± 18.0 μg/L) concentration in positive control group (G_2_) after the induction with phenylhydrazine as compared to the negative control group. Spinach-iron oxide nano particles treatment group (G_4_) showed the most effective results on serum concentrations among the different treatment groups. There was a significant increase (p ≤ 0.05) in serum iron (81.00 ± 2.73 μg/dL), serum ferritin (59.00 ± 2.73 μg/L) and TS (22.2 ± 2.68 %) levels and a significant decrease (p ≤ 0.05) in serum transferrin and TIBC (363.00 ± 49.5 μg/L) concentration in Spinach-iron oxide nanoparticle group when compared to other anemia induced groups (G_2_, G_3_, G_5_).

Though better in G_4_, these results were comparable with ferrous sulfate supplement group (G_5_), which also showed improvement in iron deficiency anemia and concentrations of serum iron, serum ferritin and transferrin saturation were found to increase whereas, a significant decrease in serum transferrin (12.8 ± 2.28 %) and TIBC (391.78 ± 64.4 μg/L) in comparison with positive control group was observed. The line in Fig. 7 showed the transferrin saturation % (TS%) within different groups. The closest TS% to the negative control group has been shown by G_4_ (22.2 %), while G_2_ experienced the lowest TS% (4.01 %) of all groups, indicating iron deficiency anemia. Here also better results were observed for G_4_ even in comparison to the iron supplement group (G_5_) (12.8 %). When compared to the positive control group (G_2_) there has been no significant difference (p ≥ 0.05) in the relative concentrations of serum iron, serum ferritin, serum transferrin, TIBC and TS in spinach powder group (G_3_) showing no positive impact.

### 3.7 Liver function tests

Table 2 describes the levels of aspartate aminotransferase (AST), alanine aminotransferase (ALT), total bilirubin, direct bilirubin and indirect bilirubin in all the groups. The results indicated that the levels of AST (45.22 ± 10.10 U/L), ALT (65.55 ± 8.81 U/L), total bilirubin (1.32 ± 0.14 md/dL), direct bilirubin (0.38 ± 0.16 mg/dL) and indirect bilirubin (0.83 ± 0.39 mg/dL) were the highest in positive control group showing damage to the liver due to induction with phenylhydrazine and were significantly different (p ≤ 0.05) from all the other groups representing iron deficiency anemia. All the groups with treatment for iron deficiency anemia showed that, irrespective of the treatment, all groups depicted the normal range of indirect bilirubin, total bilirubin, AST, ALT and direct bilirubin (Table 2). However, it can be observed that lowest values (near to negative control group) were observed in G_4_ given nanoparticles-based treatment even as compared to G_5_, provided with pharmaceutical iron supplement.

### 3.8 Renal function tests

A significant increase (p ≤ 0.05) in values of creatinine (1.57 ± 0.31 md/dL) and blood urea nitrogen (21.33 ± 6.67 mg/dL) in positive control group were observed, which indicated a disturbance in renal function tests after iron deficiency anemia induction. Comparatively, all the other treatment groups showed no significant difference in the concentrations of creatinine and BUN when compared to the negative control (normal) group with a concentration of creatinine and BUN at 0.77 ± 0.13 mg/dL and 13.33 ± 5.63 mg/dL respectively (Table 2). Furthermore, it can also be observed that though not significantly but creatinine and BUN concentrations are even lower in G_4_ (0.86 ± 0.20 and 14.33 ± 3.50 mg/dL respectively) as compared to G_5_ (0.90 ± 0.22 and 16.44 ± 2.65 mg/dL respectively).

## 4 Discussion

The present study was conducted to synthesize iron oxide NPs by green synthesis from spinach and to determine the effect on iron deficiency anemia in comparison with ferrous sulfate and spinach powder (Elshemy, 2018). In this study iron oxide nanoparticles were synthesized through spinach leaves extract when Iron (III) nitrate 9-hydrate salt was added to the extract as a reducing agent (Obidah Abert et al., 2022; Di Iorio et al., 2019). Characterizations such as FT-IR, SEM, XRD and UV–vis spectra showed the formation of spinach based iron oxide nanoparticles, while FTIR confirmed the hydroxyl and amid groups presence. From these IR spectra, it is apparent that the hydroxyl and amino groups found in proteins and carbohydrates exhibited the capability to serve dual roles in both reducing and stabilizing iron oxide nanoparticles. The current peaks and groups analysis are comparable to the previous study (Fayyaz et al., 2022; Tyagi et al., 2021; Hashem et al., 2018). The existence of Spinach-iron oxide NPs was revealed by XRD patterns with (110), (200) and (211) planes with body-centered cube crystalline structure that are in accordance with a previous study, which also observed a similar response for the produced nanoparticles. Similarly, SEM analysis also confirmed the presence of iron oxide NPs (Fayyaz et al., 2022; Murgueitio et al., 2016). The topographical view of the micrograph showed that iron oxide nanoparticles were aggregated and varied in size and shape in accordance with the previous research study (Obidah Abert et al., 2022). The UV–vis spectrum from ∼225 to ∼ 275 nm showed a broad absorption band which was observed throughout the spectral range. The optical band gap of spinach-iron oxide nano particles was estimated to be 3.08 eV, calculated by using the Tauc relation, where the absorption band was around ∼350 nm corresponding to the gap of magnetite that is in agreement with previous studies (Dhanaraj et al., 2020; Abdullah et al., 2020).

Induced iron deficiency anemia using phenylhydrazine deviates the blood parameters including haemoglobin, RBCs, HCT, MCV and MCHC (Hegde and Puranik, 2020). In the present bio-evaluation study, spinach-iron oxide nanoparticles administered induced a significant increase in blood haemoglobin concentration. The effect of green synthesized iron oxide nanoparticles is similar to a previous study conducted by Hashem et al. (2018) with the aim to examine the chemically synthesized iron oxide nanoparticles on blood haemoglobin concentration in iron deficiency anemia in rats at different levels for the duration of 4 weeks. This demonstrates that green synthesis is also effective as chemical synthesis however, former is more sustainable, ecofriendly and cost effective. Furthermore, the effect of iron oxide nanoparticles on other blood markers such as MCV, RBCs and MCHC in the current study is similar to the one reported in a previous study by El-Bahr et al. (2021), which examined the effect of biosynthesized iron oxide NPs on the blood parameters in lead-acetate induced anemia in male albino rats.

The results after the treatment plan of Spinach-iron oxide nanoparticles, either chemically or green synthesized, showed positive effect on the blood profile of iron deficiency anemia rats, however in chemical synthesis there is always a danger of toxic side effects on body as well as environment. Moreover, not only haemoglobin but also MCV, MCHC and RBCs have shown a significant increase in the concentrations. Likewise, Elshemy (2018) reported significant differences in the levels of RBCs, Hb and HCT in the supplement group when assessed with the anemia group, even though the values are within normal range or close to the normal ranges. But the group that had been treated with chemically synthesized iron oxide nanoparticles showed more significant effect on RBCs, Hb, HCT, MCV and MCHC levels than ferrous sulfate treated group. Similarly, in present study, the improved hematological parameters indicate that iron bioavailability has been enhanced by spinach iron oxide nanoparticles (Elshemy, 2018; El-Bahr et al., 2021; Hashem et al., 2018).

The current study also investigated the effect of Spinach-iron oxide nanoparticles parameters including ferritin, TS, TIBC, serum iron, and serum transferrin. A research study done by Elshemy (2018) compared the impact of ferrous sulfate and iron oxide nanoparticles on different blood parameters including ferritin, TS, TIBC and serum iron of iron deficiency anemic male albino rats. It concluded that both the iron supplement and iron oxide nanoparticles showed positive results of these parameters when compared to the anemia group; additionally, there was a significant increase of transferrin saturation in both iron oxide NP and ferrous sulfate groups when assessed with anemia group and these values have almost no or very little significant difference when compared to the control group (Elshemy, 2018). This indicated that spinach-based iron nanoparticles may have enhanced the iron absorption and bioavailability of oral iron (El-Bahr et al., 2021; Hu et al., 2004). A similar result had been reported by Shafie et al. (2016), who described the effect of single and double doses of ferrous sulfate and iron oxide nano particles on the blood and inflammatory markers in anemic rats. The results showed that the treatment group with single dose of nanoparticle had significantly different levels of TS and serum ferritin concentration as compared to other treatment groups. Additionally, there have been significantly different levels of TIBC observed in the single dose treatment of nano particles than any other group. Whereas, in the case of serum iron, the group with treatment of double dose of nano material showed most significant results when compared to other treatments. In the present study the slightly elevated levels of AST, ALT and other liver functions in the diseased group may indicate the hepatic damage (Ibrahim et al., 2012). Smital et al. (2020) conducted a research study on the purpose of checking the toxicity of green synthesized iron oxide nano formulation on various parameters including liver function tests. The results indicated that there has been no significant effect on any of the liver function test in the treatment groups of low and medium dose at 14th day, but a slight increase had been observed in the treatment group of medium dose at 28th day. Treatment group having high dose resulted in significant increase in the levels of direct and indirect bilirubin, depicting that low doses as in our study tend to be safe for liver. Recent research by Pereira et al. (2015) makes it absolutely evident that iron oxide nanoparticles provide people with safe and well-absorbed iron. Similarly, the present study shows that the administration of Spinach-iron oxide nano particles in rats had no significant effect in any of the liver function test; this decrease in the treatment groups may indicate the improvement in hepatic damage.

In order to assess renal function, the current research investigation examined blood urea nitrogen and creatinine where the elevated levels of creatinine and BUN may indicate renal impairment**.** The study done by Smital et al. (2020) indicated that there has been no significant effect on any of the renal function test in the treatment groups of low and medium dose at 14th day. Similar effects were observed in the present study where, normal levels of renal function tests in treated rats may indicate that all the groups irrespective of treatment showed no toxic effect on renal function thus managed to normalize the kidney function compared to no treatment group.

## 5 Conclusion

The major purpose of the present study was to address the long-standing limitations of traditional iron supplementation i.e. mainly poor bioavailability and gastrointestinal intolerance, by employing a cost-effective and eco-friendly biosynthetic strategy. Our study showed that spinach extract assisted iron oxide nanoparticles were significantly more effective than traditional iron supplementation that increased hemoglobin and serum ferritin levels by 12 g/dL and 59 μg/L, respectively, after 21 days of therapy, compared to usual iron supplement (11 g/dL and 55 μg/L respectively). Moreover, these nanoparticles, made through an eco-friendly, one-step green synthesis process, have higher solubility and bioavailability due to their nanoscale size, therefore promoting better absorption and systemic iron utilization. These results indicate that spinach-mediated iron oxide nanoparticles offer a viable, long-lasting, and superior alternative to traditional iron therapy for the treatment of iron deficiency anemia. However, further studies are needed to confirm long-term safety and evaluate translational potential in human subjects.

## Credit author statement

Conceptualization, A.T. and H.U.; validation, H.U. and M.A.R.; resources, M.A.R., E.Z., H.U., and M.F.R.; software, E.Z. and S.A.; investigation, S.A.; formal analysis, M.U. and Z.Q.; data curation M.A.R., R.N., and H.U.; writing—original draft preparation, M.U. and A.T.; writing—review and editing, M.A.R., M.F.R., M.A.A., S.P.A. and E.Z.; visualization, M.A.R. and H.U.; supervision, H.U.; funding, M.F.R. M.A.A. and E.Z. Project administration, M.U., M.A.A., and M.A.R. All authors have read and agreed to the published version of the manuscript.

## Consent for publication

All authors agreed for publication of this manuscript.

## Code availability

Not applicable.

## Ethical approval

The experimental procedure was approved by the Animal Ethical Committee of Government College University, Faisalabad, Punjab, Pakistan.

## Availability of data and material

The data generated during this study will be made available upon request from the first author.

## Funding

This research was funded by 10.13039/501100006261Taif University, Taif, Saudi Arabia project No.(TU-DSPP-2025-02).

## Declaration of competing interest

The authors declare that they have no known competing financial interests or personal relationships that could have appeared to influence the work reported in this paper.
